# Gender Differences in the Quality of Life of Formal Workers

**DOI:** 10.3390/ijerph18115951

**Published:** 2021-06-01

**Authors:** José Andrade Louzado, Matheus Lopes Cortes, Marcio Galvão Oliveira, Vanessa Moraes Bezerra, Sóstenes Mistro, Danielle Souto de Medeiros, Daniela Arruda Soares, Kelle Oliveira Silva, Clávdia Nicolaevna Kochergin, Vivian Carla Honorato dos Santos de Carvalho, Welma Wildes Amorim, Sotero Serrate Mengue

**Affiliations:** 1 Multidisciplinary Health Institute, Federal University of Bahia, Vitória da Conquista 45029-094, Brazil; matheuscortes@hotmail.com (M.L.C.); mgalvao@ufba.br (M.G.O.); vanessaenut@gmail.com (V.M.B.); smistro@gmail.com (S.M.); daniellesoutomedeiros@gmail.com (D.S.d.M.); dandani23@yahoo.com.br (D.A.S.); kelle.oliveira@gmail.com (K.O.S.); kochergin62@gmail.com (C.N.K.); vihonorato@hotmail.com (V.C.H.d.S.d.C.); 2 Department of Natural Sciences, State University of Southwest of Bahia, Vitória da Conquista 45083-900, Brazil; welma.wilde@uesb.edu.br; 3 Graduate Program in Epidemiology, School of Medicine, Federal University of Rio Grande do Sul, Porto Alegre 90035-002, Brazil; sotero@ufrgs.br

**Keywords:** quality of life, worker health, worker categories, gender, health

## Abstract

Background: This study aimed to assess the quality of life associated with gender inequalities in formal workers and to determine the effect of sociodemographic, clinical, and behavioral factors on the quality of life (QOL). Methods: This cross-sectional study involved 1270 workers. Quality of life was measured using the EUROHIS-QOL 8-Item and assessed in terms of psychological, environmental, social, and physical domains, while demographic, socioeconomic, behavioral, and clinical variables served as explanatory variables. Analyses were performed using an ordinal logistic regression model whose significance level was 5%. Results: Of the participants, 80.2% were men, and 19.8% were women; the mean age was 34 (standard deviation: ±10) and 32 (±9) years, respectively. In all prediction scenarios, men were more likely to have a higher quality of life, especially in the physical (odds ratio: 2.16; 95% confidence interval: 1.60–2.93) and psychological (odds ratio: 2.09; 95% confidence interval: 1.51–2.91) domains. Conclusions: Men and women had significantly different levels of quality of life, and sociodemographic, clinical, and behavioral variables partially clarified these differences, which were possibly established by a socio-historical process of construction of the work role determined by gender issues.

## 1. Introduction

The differences between men and women are not restricted to the biological field, as gender inequalities exist in economic, social, political, and labor fields. The unique characteristics of men and women, which should complement each other, have, throughout history, become factors that promote gender inequality and, consequently, unequal health and quality-of-life (QOL) conditions among workers [1,2].

QOL has a complex conceptual framework that comprises several dimensions relating to individuals’ self-perception of their life condition (i.e., the paradigms through which individuals view the world and plan their life); specifically, it features physical, psychological, social, and environmental dimensions. Several instruments have been developed for measuring QOL based on this concept, including the EUROHISQOL 8-Item, which was created by selecting the most representative items of the WHOQOL-BREF domains. The EUROHISQOL 8-Item is a simplified version of the WHOQOL-BREF domains but is easier to apply and has lower application costs; further, it retains all psychometric properties of the original instrument [3,4].

Analysis of QOL data obtained through the EUROHISQOL 8-Item must include consideration of the context and peculiarities of the studied population. Among formal workers (characterized mainly by the link established between employer and employee, with a relationship mediated by an employment contract that establishes the rights and duties determined in the labor legislation), QOL is influenced by work relationships and sociodemographic, clinical, and behavioral variables. However, characteristics such as gender can also have important unique influences; historically, gender has influenced the access to and roles of men and women in the labor market [2,5].

In Brazil, gender inequalities are reflected in various aspects of the labor market. The average weekly workload for men is 41 h and 37.8 h for women. When stratifying the hours of work, it is observed that 83% of unpaid activities are performed by women, while men (61.2%) are more prevalent in paid activities [6]. This division of labor results in an economic imbalance caused by gender issues in the social division of labor and makes women more prone to physical and mental illness, which significantly compromises their QOL levels [2].

Another important aspect of gender inequalities in the Brazilian population is evidenced when analyzing the type of occupation and salary. Women usually occupy positions of low social appreciation and of lower remuneration. These differences are even more significant when they are compared to men in the same working conditions and with similar productive characteristics but with better salaries [5].

Understanding gender inequalities is essential for understanding how people access the labor market and the associated impacts on one’s health condition. In this regard, existing findings concerning economic and health indicators already suggest that women professionals experience unfavorable health situations when compared to men, and in most cases, this disadvantage among women cannot be explained solely by clinical and biological variables. In this context, QOL can be a good indicator in analyzing the health situation of formal workers, as it can help in identifying individual and collective health needs that should be prioritized for the planning and resource allocation to mitigate the challenges in the area of occupational health [7,8].

The population of formal workers in Brazil has hardly been explored in the context of health-focused studies despite its size and economic representativeness (47,554,211 people, who generated a salary mass of approximately R$ 1.8 trillion in 2019). In 2019, 56% of this group of workers had a median salary of R$ 1859.18. Of this 56%, just 44% were women; the median salary for these women was R$ 1642.95, 11.63% less than that of men [9,10].

Identifying QOL among workers and possible causes of gender inequalities concerning health may contribute to improvements in health planning. The data obtained from such analyses may help planners develop policies that sufficiently account for specific gender peculiarities, such as differences regarding productivity, occupational access, and discrimination, all of which are factors that contribute to physical and mental illness [2,5,11]. This study aimed to assess the quality of life associated with gender inequalities in formal workers and to determine the effect of sociodemographic, clinical, and behavioral factors on the predictive analysis model.

## 2. Methods

### 2.1. Study Design and Population

This is a cross-sectional study, integrated with the HealthRise Vitória da Conquista cohort. The HealthRise program focused on patients with chronic diseases (specifically, hypertension and diabetes) and was designed to improve, for such patients, access to health services, quality care, and electronic medical records and increase the availability of special tests for the diseases in question [12].

The municipality of Vitória da Conquista is located in northeastern Brazil and has a territorial area of over 3,254,000 km^2^; in 2019, its estimated population was 338,000 [13]. Most of its population (approximately 87%) lives in the urban area of the municipality [13]. The municipality is located at an important road junction for the flow of production between southeastern and northeastern Brazil and has a service-based economy that is mainly associated with the sectors of health, education, trade, and construction.

### 2.2. Data Collection

Data were collected at Serviço Social da Indústria (SESI), a private, non-profit entity whose mission is to provide professional qualifications and promote worker health [14]. The study sample comprised a population of workers who were receiving assistance from SESI, and data were collected between August 2017 and July 2018. The inclusion criteria for the sample were being 18 years of age or older, living in the municipality, and attending SESI for periodic consultations with an occupational physician. The exclusion criteria were living in another municipality and awaiting a pre-dismissal medical evaluation.

In total, 2014 workers fulfilled the inclusion criteria and constituted the study sample universe. For the sample calculation, a 95% confidence level was used with a 50% prevalence (due to the multiple outcomes measured in the main project) and a tolerable error of 2%. The final sample, after considering a loss of 10%, included 1218 workers. Thus, after applying the selection criteria, 1270 subjects remained as participants in this research.

### 2.3. Procedure

Data were collected by trained interviewers (undergraduate medical students) who conducted the interviews using a digital questionnaire; the questionnaire was administered on tablets through the KoBoToolbox platform. The questionnaire was adapted from the Brazilian National Health Survey 2013 [15] and also collected other information relevant to the research focus, such as self-reported assessments of self-care among patients with chronic diseases (hypertension and diabetes), level of access to health services, and stress and QOL levels.

The objective measures collected were weight and height. Weight was measured while individuals were barefoot and were wearing light clothing; the SECA 813^®^ digital portable electronic scale, duly calibrated, was used. A portable NutriVida^®^ stadiometer was used to measure height, during which individuals were barefoot and in an upright position.

### 2.4. Instruments and Measurement Variables

QOL was considered an outcome variable and was measured using the EUROHIS-QOL 8-Item, an instrument created by the WHO. To improve the comparison of data between countries, the WHO endeavored to develop measurement tools for health indicators that accommodate the unique economic characteristics and administration requirements of different countries [3,4].

The EUROHIS-QOL 8-Item, which has been translated into Brazilian Portuguese and has been validated, comprises the four domains of the WHOQOL-BREF: physical, psychological, social relations, and environment; the EUROHIS-QOL 8-Item also features a general, global domain. All eight items of the EUROHIS-QOL 8-Item are answered using a five-point Likert scale, with scores ranging from 1 to 5 for each item.

In this research, the comparisons between men and women were based on the total QOL scores for each domain; these were treated continuously and categorized into tertiles in which higher scores indicated higher QOL. The eight items of the EUROHIS-QOL 8-Item are distributed across its domains as follows: two general items relate to the global domain (“How would you evaluate your life?” and “How satisfied are you with your health?”); two items relate to the physical domain (“Do you have enough energy in your day-to-day life?” and “How satisfied are you with your ability to perform day-to-day activities?”); one item concerns the psychological domain (“How satisfied are you with yourself?”); one item concerns the social-relations domain (“How satisfied with your personal relationships: friends, relatives, acquaintances, and colleagues?”); and two items concern the environment domain (“Do you have enough money to meet your needs?” and “How satisfied are you with the conditions of the place where you live?”).

Demographic, socioeconomic, behavioral, and clinical variables were considered explanatory variables. The Brazilian Economic Classification Criterion (Critério de Classificação Econômica Brasil) of the Brazilian Association of Research Companies (Associação Brasileira de Empresas de Pesquisa), which was enacted in 2015 and updated in terms of class distribution in 2016, was used to quantify socioeconomic status [16]. Only two categories were included for marital status: living with a partner or not living with a partner. The working schedule was also divided into two categories: working exclusively during the day and working other schedules.

The behavioral variables measured included diet, smoking status, alcohol consumption, and physical activity. A healthy diet was defined as the consumption of at least one portion of fruit or fruit juice and two portions of vegetables and/or legumes at least five times a week [15,17]. Workers who reported any frequency of tobacco use, even sporadic, were considered smokers [15]. Among women, high-risk alcohol consumption was characterized as the ingestion of four or more doses on the same occasion within the past 30 days; among men, high-risk alcohol consumption was defined as the ingestion of five or more doses on a single occasion [15]. Finally, based on the standard used by the International Physical Activity Questionnaire, individuals who performed more than 150 min of physical activity per week were considered to be physically active [18,19].

Clinical variables were self-reported health [15] (three levels: “very good,” “good,” and “regular, poor, and very poor”) and nutritional status; this was classified using body mass index (BMI; calculated as weight/height^2^) and grouped into two levels: non-obese (BMI ≤ 29.9 kg/m^2^) and obese (BMI ≥ 30 kg/m^2^) [20,21,22,23].

### 2.5. Statistical Analysis

First, descriptive analysis of the data was performed using absolute numbers and percentages. Continuous variables were presented using means, and categorical variables were presented as simple frequencies and percentages. The homogeneity of variance of means was evaluated by Levene’s test. ANOVA or Brown–Forsythe tests were used to determine the differences between means, and a Tukey’s HSD test was used to show the differences. A two-way ANOVA was then used to compare mean QOL scores across men and women with different socioeconomic and clinical characteristics.

Stepwise ordinal logistic regression with cumulative probabilities and proportional odds was used to determine the effect of risk behaviors, clinical conditions, sociodemographic characteristics, and economic determinants on QOL levels in terms of each domain; a level of significance of 20% (*p* < 0.20) was applied for the explanatory variables and their theoretical aspects. The proportional odds were assessed using the total likelihood ratio, comparing the adjusted models with models with variable location parameters. A goodness-of-fit test indicated that the model had a good fit for the observed data and statistically significantly predicted the dependent variable. Logistic regression generated estimates that were analyzed using 95% confidence intervals and a significance level of 5%. Statistical analyses were performed using IBM SPSS Statistics version 26.0.

### 2.6. Ethical Aspects

This research was approved by the Research Ethics Committee of the Federal University of Bahia/Multidisciplinary Health Institute—Campus Anísio Teixeira (CAEE number 62259116.0.0000.5556). All workers involved provided written informed consent.

## 3. Results

The results confirm the existence of persistent gender inequality in the QOL levels, even after developing the analysis model and identifying the effect of the sociodemographic, clinical, and behavioral explanatory variables. When considering the QOL domains, the objectives of this study are clearly demonstrated by the persistent inequalities between men and women in most of the dimensions of the outcome variable that were analyzed.

Gender inequalities in the insertion in the labor market were evidenced by the results of the study, showing that women with a similar average age to men occupied fewer job positions. The total number of workers who participated in this study was 1270, of whom 80.2% were men and 19.8% were women. Their average age was 34 years (standard deviation: ±10) and 32 years (±9), respectively.

Most men (51.7%) were in economic class C, D, or E. They were either married or cohabiting (65.1%), had brown skin color (57.2%), and had very good or good self-reported health (66.4%). Meanwhile, most women (48.6%) were in economic class A or B, were married or cohabiting (50.4%), had brown skin color (64.5%), and had very good or good self-reported health (55.4%). The prevalence of obesity was higher among women (19.8%) than men (13.8%). Proportions of tobacco use (6.3) and high-risk alcohol consumption (2.1) were both higher among men (Table 1).

The results also showed important differences between men and women for almost all domains of the QOL construct, and the effect of the explanatory variables was not observed proportionally in both sexes. Although important differences were observed between men and women for the psychological, social, physical, and global domains (*p* < 0.05 for all domains), no statistically significant gender differences were observed for the environmental domain. When considering all workers together (men and women), the only explanatory variable that showed any statistically significant difference was self-reported health (Table 2).

When considering each QOL domain, significant differences between men and women were observed for certain explanatory variables. In the physical domain, men showed higher mean QOL for all explanatory variables (*p* < 0.05); for the psychological and global domains except for smoking, men showed higher mean QOL for all variables (*p* < 0.05); in the social domain, men from social classes A and B (4.27) who had black skin color (4.19), who had brown skin color (4.19), who worked in the morning (4.20), who were obese (4.22), who ate healthy food (4.19), who had high-risk alcohol consumption (4.22), and who were physically active (4.18) showed higher mean QOL (*p* < 0.05). The environmental domain was the only domain that featured a statistically significant difference in favor of women; this was concerning nutritional status in which the highest mean QOL was associated with non-obese women (7.05). (Table 3).

The analysis model was developed for determining the effect of explanatory variables on QOL levels as well as for determining whether the discrepancies of the odds ratio (OR) measure for men and women were maintained, even after adjusting the model. In this sense, the ordinal logistic regression model showed, in the raw and adjusted odds ratios (ORs), the presence of statistically significant differences between men and women for all QOL domains except for the environmental and the social domains (models f and g, respectively).

The modeling results showed that gender inequalities were consistent since, even after identifying and determining the effect of the explanatory variables, the ORs of men and women remained discrepant. This was observed after adjusting the regression model for all explanatory variables: the ORs for men reduced by approximately 24% for the psychological domain, 10% for the physical domain, and 18% for the global domain. In all prediction scenarios produced by the models, men were more likely to have better QOL levels, especially for the physical domain (OR = 2.16; 95% CI: 1.60–2.93) and the psychological domain (OR = 2.09; 95% CI: 1.51–2.91), in which the ORs were more than double than those of women (Table 4).

## 4. Discussion

This study aimed to investigate gender inequalities and the effect of explanatory variables (clinical and behavioral sociodemographic variables) on QOL levels in formal workers. The results presented in this study corroborate other studies that have already identified gender differences in other population groups [24,25]. Thus, the main findings of this study indicate that a consistent inequality in QOL exists between male and female formal workers. When this analysis was stratified by QOL domains (psychological, environmental, social, physical, and global), such discrepancies became even more evident; further, adjusting the regression model for explanatory variables was not sufficient to clarify the important discrepancies between the respective QOL values of men and women.

The proportion of women in formal employment is lower than that of men; this was also observed among the sample for the present study. A reason for this is that, in the contemporary job market, the only personal characteristics valued are those associated with productivity, and total and unrestricted availability is expected; meanwhile, pregnancy and child-care responsibilities contribute negatively to securing a job. These factors create an employee profile that is closely associated with the male gender, which further deepens gender inequalities [26,27].

The psychological domain of QOL is mainly influenced by self-esteem, which is a subjective self-assessment that is determined by health conditions, work, and social inclusion. Work is a determining factor for an individual’s social class and lifestyle and, consequently, their access to goods and services. However, there are discrepancies between genders in this regard, with women in operational positions often receiving less than men who are at the same hierarchical level. As a result of such socially constructed and historically consolidated discrimination, to secure a space in the labor market, women must do much more than have sufficient professional qualifications. This can lead to mental illness, which decreases women’s QOL levels in the psychological domain [26,28,29,30], a finding that was also observed in this study.

In the social domain of QOL, men again showed higher levels than women. This QOL domain is directly associated with social relationships established by individuals. The common social construction in which the man is the provider and the woman the caregiver forces many women to reconcile family obligations with professional activities, mainly due to the need to help with the family budget; this inevitably exacerbates physical and mental fatigue. In contrast, for men, working in a formal job, in addition to representing social and economic value, represents dignity, capacity, and professional fulfillment [31,32,33,34]. The inequalities between men and women in this context are clearly visible in the types of jobs common to each gender, particularly in the private sector; many women secure jobs that facilitate part-time hours with a lower payment and greater precariousness [28].

The physical domain of QOL is characterized by an individual’s ability to have sufficient energy to perform activities of daily living, which involves everything from the basic needs of daily life to work activities. In this domain, men again showed a higher average QOL than women. Factors that may contribute to these differences include the higher prevalence of double working hours and child care and marriage obligations among women. These factors are associated with a shorter time for leisure and rest and, consequently, expose women to risks of musculoskeletal problems and fatigue [25,35,36].

The global domain of QOL is influenced by self-reported health, which is a variable that has a good predictive capacity for individual and collective health conditions and is also a good indicator of morbidity and mortality [37]. Self-reported health is influenced by a combination of socioeconomic, behavioral, and clinical variables [38,39]; for all of these, men showed higher QOL averages. Even when adjusted for socioeconomic class, gender inequalities persist, including among workers who have the same organizational and job-responsibility level [24,40].

The higher levels of global QOL among men may be related to how they perceive their health needs. Women have a better perception of their health conditions because, traditionally, they are responsible for the care of children and other family members when they become ill, which heightens their sensitivity to the identification of health problems. Men, on the other hand, have a different perception of illness, usually influenced by a patriarchal, historical conception of illness as a manifestation of weakness and vulnerability, incompatible with the genderist idea that “men do not get sick” [41,42,43]. Thus, analysis of this QOL indicator must consider the differing experiences and conceptions of each gender and how this impacts their respective understanding of the health and disease process.

The inequalities caused by health determinants and conditions are not sufficient to explain differences in QOL between genders, especially as many health discrepancies have gender issues as an essential cause. Consequently, a regression model was developed that could verify the effects of explanatory variables on QOL levels and clarify the potential differences caused by gender issues and the asymmetric power relations between genders.

Consequently, even after adjusting the model for age group, marital status, work regime, economic class, diet, nutritional status, smoking status, alcohol intake, self-reported health, and physical activity, in the psychological domain, men were twice as likely to have higher QOL levels than women. This lower likelihood among women may be related to the demands of double working hours, the need to maintain one’s physical appearance, exposure at work to typical male behaviors, and the need to gain institutional respect from colleagues. Endeavoring to satisfy these requirements can result in psychological distress and consequent deterioration of mental health [24,26,44].

In the physical and global domains, men were more likely to have higher QOL levels, even after adjusting the regression model for all explanatory variables. These persistent differences presented in the model indicate that, for women, the demands of the job market, such as physical and productive capacity, professional qualifications, and physical appearance, create difficulties and that existing job market demands are not suited to accommodate gender peculiarities [27,28,29].

Men were also more likely to have higher QOL levels than women in the social domain, and the adjustments made to the regression model through the explanatory variables increased the strength of this inequality between men and women. Some important issues are caused by power and subordination relationships that exist in the professional sector, such as moral and gender harassment, wage inequality, and discrimination regarding work capacity; such issues may affect women’s QOL. Notably, advances in labor legislation have not been able to reverse these gender inequalities [45,46,47].

Some methodological limitations should be taken into account when considering the results of this study. First, the study featured a cross-sectional design, which is restricted to determining associations and does not allow the identification of causal relationships; this increases the risk of reverse causality [48]. Moreover, the presence among participants of acute pathologies at the time of data collection and their possible influence on QOL levels were not checked.

The findings of this study are relevant for future research not only because this study identified an association between QOL and sociodemographic, clinical, and behavioral variables, but also because it identified important differences in QOL levels between male and female formal workers. All analyzed models showed that men were more likely to have higher QOL levels than women in the psychological, social, physical, and global domains, with the environmental domain being the only one that did not show statistically significant differences in the analysis model (ordinal logistic regression).

There were significant discrepancies in QOL levels between the male and female participants, and sociodemographic, clinical, and behavioral variables were insufficient to explain these differences. These discrepancies were possibly established by the socio-historical construction of the professional roles of genders. The results of this study may help guide the implementation of public policies in the area of occupational health; in particular, gender issues should be used as a guiding element in health planning, and efforts should be made to mitigate inequalities between men and women.

## Figures and Tables

**Table 1:** Sociodemographic, economic, clinical, and behavioral characteristics of workers in the Industrial Social Services of Vitória da Conquista.

Variables	Male	Female
	*n* (%)	*n* (%)
Total workers	1019 (80.2)	251 (19.8)
Age range		
Up to 29 years	377 (37.0)	92 (36.7)
30 to 39 years	390 (38.3)	102 (40.6)
40 to 49 years	168 (16.5)	49 (19.5)
50 years or more	84 (8.2)	8 (3.2)
Economic class **		
A + B1 + B2	343 (33.7)	122 (48.6)
C1 + C2	527 (51.7)	102 (40.6)
D + E	149 (14.6)	27 (10.8)
Marital status *		
Married or cohabiting	663 (65.1)	126 (50.4)
Single/divorced/widowed	356 (34.9)	124 (49.6)
Race/skin color *		
White	197 (20.4)	50 (21.4)
Black	216 (22.4)	33 (14.1)
Brown	551 (57.2)	151 (64.5)
Work schedule *		
Daytime	795 (79.2)	219 (88.3)
Night/day and night/on call at night	209 (20.8)	29 (11.7)
Self-reported health *		
Very good	136 (13.4)	34 (13.5)
Good	540 (53.0)	105 (41.9)
Regular/poor/very poor	342 (33.6)	112 (44.6)
Diet		
Healthy diet	411 (43.6)	112 (45.7)
Unhealthy diet	532 (56.4)	133 (54.3)
Smoking *		
No	915 (89.9)	247 (98.4)
Yes	103 (10.1)	4 (1.6)
High-risk alcohol consumption		
No	693 (68)	213 (84.9)
Yes	326 (32)	38 (15.1)
Physical activity		
Active	640 (62.8)	151 (60.2)
Not active	379 (37.2)	100 (39.8)
Nutritional status *		
Non-obese	859 (86.2)	190 (80.2)
Obese	137 (13.8)	47 (19.8)

* Variables with missing data (i.e., variables for which some participants provided no response). ** These criteria evaluate individuals’ socioeconomic level through a household assessment. Scores range from 0 to 100 points, with higher scores representing a higher economic stratum: A (45–100 points), B1 (38–44 points), B2 (29–37 points), C1 (23–28 points), C2 (17–22 points), and D/E (≤16 points).

**Table 2:** Influence of each sociodemographic, economic, and behavioral characteristic on quality-of-life domains.

Variables	Quality-of-Life Domains									
	Psychological		Environmental		Social		Physical		Global	
	QoL	*p*	QoL	*p*	QoL	*p*	QoL	*p*	QoL	*p*
Gender										
Male	4.16	0.000	6.95	0.998	4.17	0.011	8.08	0.000	7.74	0.000
Female	3.79		6.95		4.04		7.43		7.23	
Age range										
Up to 29 years	4.09	0.044	7.14	0.000	4.17	0.361	7.91	0.638	7.73	0.028
30 to 39 years	4.07		6.89		4.11		7.99		7.65	
40 to 49 years	4.05		6.64		4.13		7.88		7.42	
50 years or more	4.29		7.04		4.23		8.04		7.67	
Socioeconomic class *										
A + B1 + B2	4.05	0.251	7.39	0.000	4.22	0.029	7.94	0.934	7.66	0.516
C1 + C2	4.12		6.77		4.11		7.94		7.61	
D + E	4.08		6.44		4.08		7.98		7.73	
Marital status										
Married or cohabiting	4.12	0.045	6.88	0.013	4.16	0.447	7.98	0.338	7.66	0.561
Single/divorced/widowed	4.04		7.07		4.13		7.90		7.62	
Race/skin color										
White	4.11	0.751	7.20		4.12		7.98		7.67	
Black	4.12		6.84	0.005	4.18	0.593	8.06	0.360	7.68	0.883
Brown	4.08		6.94		4.16		7.92		7.64	
Work schedule										
Daytime	4.10	0.826	6.94	0.378	4.17	0.091	7.95	0.947	7.66	0.280
Night/day and night/on call at night	4.08		7.02		4.08		7.94		7.56	
Self-reported health										
Very good	4.36	0.000	7.28	0.000	4.32	0.000	8.54	0.000	8.68	0.000
Good	4.19		7.09		4.19		8.16		7.86	
Regular/poor/very poor	3.85		6.63		4.02		7.43		6.95	
Nutritional status										
Non-obese	4.14	0.000	6.94	0.363	4.15	0.990	8.01	0.001	7.75	0.000
Obese	3.79		7.04		4.15		7.66		7.00	
Diet										
Healthy diet	4.11	0.573	7.02	0.078	4.16	0.549	7.98	0.381	7.64	0.961
Unhealthy diet	4.08		6.88		4.13		7.92		7.64	
Smoking										
Yes	4.08	0.923	6.60	0.004	4.05	0.124	8.06	0.384	7.64	0.999
No	4.09		6.98		4.16		7.94		7.64	
High-risk alcohol consumption										
Yes	4.13	0.178	6.99	0.468	4.20	0.074	8.14	0.002	7.77	0.018
No	4.07		6.93		4.12		7.87		7.59	
Physical activity										
Active	4.13	0.026	6.96	0.793	4.15	0.858	7.99	0.112	7.78	0.000
Non-active	4.03		6.94		4.14		7.87		7.42	

* These criteria evaluate individuals’ socioeconomic level through a household assessment. Scores range from 0 to 100 points, with higher scores representing a higher economic stratum: A (45–100 points), B1 (38–44 points), B2 (29–37 points), C1 (23–28 points), C2 (17–22 points), and D/E (≤16 points).

**Table 3:** Mean quality-of-life scores, stratified by gender and domains, for each sociodemographic, economic, and behavioral characteristic.

Variables	Quality-of-Life Domains														
	Psychological			Environmental			Social			Physical			Global		
	Men	Women	*p*	Men	Women	*p*	Men	Women	*p*	Men	Women	*p*	Men	Women	*p*
Age range															
Up to 29 years	4.16	3.82	Gd = 0.001 Age = 0.626 Gd * Age = 0.629	7.13	7.17	Gd = 0.454 Age = 0.001 Gd * Age = 0.687	4.19	4.10	Gd = 0.109 Age = 0.278 Gd * Age = 0.178	8.03	7.43	Gd = 0.000 Age = 0.946 Gd * Age = 0.558	7.81	7.40	Gd = 0.000 Age = 0.017 Gd * Age = 0.222
30 to 39 years	4.16	3.73		6.88	6.96		4.16	3.93		8.15	7.39		7.75	7.25	
40 to 49 years	4.11	3.85		6.66	6.56		4.11	4.19		7.98	7.54		7.54	7.00	
50 years or more	4.33	3.88		7.08	6.63		4.25	4.00		8.13	7.13		7.80	6.38	
Socioeconomic class *															
A + B1 + B2	4.17	3.69	Gd = 0.000 SC = 0.269 Gd * SC = 0.122	7.41	7.34	Gd = 0.419 SC = 0.000 Gd * SC = 0.867	4.27	4.07	Gd = 0.007 SC = 0.101 Gd * SC = 0.387	8.14	7.37	Gd = 0.000 SC = 0.990 Gd * SC = 0.536	7.77	7.35	Gd = 0.000 SC = 0.201 Gd * SC = 0.534
C1 + C2	4.17	3.86		6.80	6.64		4.13	4.06		8.03	7.48		7.71	7.08	
D + E	4.11	3.93		6.45	6.41		4.11	3.89		8.07	7.48		7.81	7.30	
Marital status															
Married or cohabiting	4.19	3.78	Gd = 0.000 MS = 0.580 Gd * MS = 0.499	6.88	6.90	Gd = 0.699 MS = 0.090 Gd * MS = 0.550	4.18	4.03	Gd = 0.016 MS = 0.950 Gd * MS = 0.589	8.10	7.34	Gd = 0.000 MS = 0.555 Gd * MS = 0.221	7.74	7.22	Gd = 0.000 MS = 0.873 Gd * MS = 0.896
Single/divorced/widowed	4.12	3.79		7.09	7.00		4.15	4.06		8.04	7.52		7.74	7.25	
Race/skin color															
White	4.13	4.02	Gd = 0.000 R/Cr = 0.180 Gd * Cr = 0.039	7.25	7.02	Gd = 0.365 R/Cr = 0.070 Gd * Cr = 0.259	4.16	3.96	Gd = 0.050 R/Cr = 0.361 Gd * Cr = 0.484	8.02	7.82	Gd = 0.000 R/Cr = 0.225 Gd * Cr = 0.036	7.71	7.48	Gd = 0.000 R/Cr = 0.486 Gd * Cr = 0.258
Black	4.19	3.70		6.86	6.67		4.19	4.18		8.19	7.24		7.77	7.09	
Brown	4.17	3.75		6.91	7.03		4.19	4.04		8.08	7.35		7.76	7.19	
Work schedule															
Daytime	4.18	3.79	Gd = 0.000 WS = 0.888 Gd * WS = 0.522	6.94	6.91	Gd = 0.615 WS = 0.262 Gd * WS = 0.472	4.20	4.06	Gd = 0.020 WS = 0.097 Gd * WS = 0.661	8.09	7.42	Gd = 0.000 WS = 0.842 Gd * WS = 0.700	7.76	7.27	Gd = 0.000 WS = 0.053 Gd * WS = 0.262
Night/day and night/on call at night	4.12	3.83		7.00	7.17		4.11	3.90		8.01	7.45		7.66	6.86	
Self-reported health															
Very good	4.44	4.03	Gd = 0.000 SRH = 0.000 Gd * SRH = 0.193	7.27	7.32	Gd = 0.668 SRH = 0.000 Gd * SRH = 0.430	4.34	4.24	Gd = 0.051 SRH = 0.000 Gd * SRH = 0.453	8.68	7.97	Gd = 0.000 SRH = 0.000 Gd * SRH = 0.014	8.74	8.47	Gd = 0.000 SRH = 0.000 Gd * SRH = 0.610
Good	4.23	3.99		7.07	7.24		4.20	4.15		8.21	7.92		7.92	7.52	
Regular/poor/very poor	3.95	3.52		6.65	6.57		4.07	3.88		7.63	6.79		7.08	6.58	
Nutritional status															
Non-obese	4.20	3.91	Gd = 0.000 Ns = 0.000 Gd * Ns = 0.005	6.92	7.05	Gd = 0.019 Age = 0.316 Gd * Ns = 0.001	4.17	4.06	Gd = 0.004 Age = 0.577 Gd * Ns = 0.174	8.12	7.49	Gd = 0.000 Age = 0.016 Gd * Ns = 0.989	7.83	7.40	Gd = 0.000 Age = 0.000 Gd * Ns = 0.159
Obese	3.96	3.28		7.22	6.50		4.22	3.93		7.82	7.20		7.19	6.43	
Diet															
Healthy diet	4.18	3.84	Gd = 0.000 Dt = 0.362 Gd * Dt = 0.546	7.04	6.94	Gd = 0.964 Dt = 0.422 Gd * Dt = 0.285	4.19	4.04	Gd = 0.013 Dt = 0.836 Gd * Dt = 0.597	8.16	7.32	Gd = 0.000 Dt = 0.761 Gd * Dt = 0.058	7.80	7.05	Gd = 0.000 Dt = 0.164 Gd * Dt = 0.015
Unhealthy diet	4.16	3.76		6.86	6.96		4.15	4.05		8.01	7.53		7.70	7.40	
Smoking															
Yes	4.10	3.75	Gd = 0.050 Smk = 0.759 Gd * Smk = 0.921	6.60	6.50	Gd = 0.845 Smk = 0.213 Gd * Smk = 0.917	4.05	4.00	Gd = 0.601 Smk = 0.615 Gd * Smk = 0.796	8.14	6.00	Gd = 0.000 Smk = 0.043 Gd * Smk = 0.226	7.67	7.00	Gd = 0.068 Smk = 0.620 Gd * Smk = 0.814
No	4.17	3.79		6.99	6.96		4.19	4.04		8.07	7.45		7.75	7.24	
High-risk alcohol consumption															
Yes	4.18	3.74	Gd = 0.000 Alc = 0.788 Gd * Alc = 0.538	7.03	6.71	Gd = 0.341 Alc = 0.488 Gd * Alc = 0.113	4.22	4.03	Gd = 0.027 Alc = 0.678 Gd * Alc = 0.469	8.22	7.42	Gd = 0.000 Alc = 0.409 Gd * Alc = 0.373	7.83	7.26	Gd = 0.000 Alc = 0.468 Gd * Alc = 0.678
No	4.16	3.80		6.92	7.00		4.15	4.05		8.01	7.43		7.70	7.23	
Physical activity															
Active	4.19	3.85	Gd = 0.000 Pa = 0.035 Gd * Pa = 0.483	6.98	6.87	Gd = 0.741 Pa = 0.483 Gd * Pa = 0.127	4.18	4.01	Gd = 0.027 Pa = 0.538 Gd * Pa = 0.234	8.14	7.37	Gd = 0.000 Pa = 0.841 Gd * Pa = 0.104	7.88	7.33	Gd = 0.000 Pa = 0.001 Gd * Pa = 0.419
Non-active	4.12	3.70		6.90	7.08		4.15	4.10		7.97	7.51		7.50	7.09	

Pa, physical activity; * These criteria evaluate individuals’ socioeconomic level through a household assessment. Scores range from 0 to 100 points, with higher scores representing a higher economic stratum: A (45–100 points), B1 (38–44 points), B2 (29–37 points), C1 (23–28 points), C2 (17–22 points), and D/E (≤16 points).

**Table 4:** Raw and adjusted odds ratios for male and female formal workers regarding quality-of-life scores, stratified by domains and adjusted using the ordinal logistic regression model for socio-demographic, economic, behavioral, and clinical variables.

Model Adjustment	Quality of Life Domains									
	Psychological		Environmental		Social		Physical		Global	
	OR (95% CI)	β	OR (95% CI)	β	OR (95% CI)	β	OR (95% CI)	β	OR (95% CI)	β
Not adjusted										
Women	1		1		1		1		1	
Men	2.76 (2.07–3.68)	1.014	1.02 (0.79–1.32)	0.018	1.34 (1.02–1.78)	0.296	2.41 (1.86–3.13)	0.879	2.15 (1.61–2.88)	0.767
Model ^a^										
Women	1		1		1		1		1	
Men	2.42 (1.79–3.28)	0.885	1.22 (0.92–1.61)	0.196	1.46 (1.08–1.97)	0.379	2.55 (1.93–3.37)	0.935	2.07 (1.51–2.82)	0.726
Model ^b^										
Women	1		1		1		1		1	
Men	2.41 (1.77–3.30)	0.882	1.21 (0.91–1.61)	0.194	1.45 (1.07–1.97)	0.371	2.54 (1.91–3.37)	0.931	2.11 (1.54–2.90)	0.748
Model ^c^										
Women	1		1		1		1		1	
Men	2.27 (1.65–3.12)	0.819	1.26 (0.94–1.68)	0.232	1.46 (1.06–1.99)	0.378	2.50 (1.87–3.34)	0.916	2.02 (1.45–2.80)	0.701
Model ^d^										
Women	1		1		1		1		1	
Men	2.31 (1.67–3.18)	0.836	1.29 (0.97–1.74)	0.258	1.49 (1.09–2.05)	0.402	2.47 (1.84–3.31)	0.904	2.03 (1.46–2.82)	0.709
Model ^e^										
Women	1		1		1		1		1	
Men	2.26 (1.63–3.13)	0.815	1.28 (0.95–1.72)	0.249	1.41 (1.02–1.94)	0.343	2.37 (1.76–3.19)	0.862	1.93 (1.39–2.70)	0.659
Model ^f^										
Women	1		1		1		1		1	
Men	2.08 (1.50–2.89)	0.734	1.21 (0.90–1.64)	0.194	1.34 (0.97–1.85)	0.289	2.16 (1.60–2.92)	0.770	1.77 (1.26–2.48)	0.569
Model ^g^										
Women	1		1		1		1		1	
Men	2.09 (1.51–2.91)	0.739	1.21 (0.90–1.64)	0.194	1.34 (0.97–1.85)	0.290	2.16 (1.60–2.93)	0.772	1.77 (1.25–2.49)	0.569

CI, confidence interval. Ordinal logistic regression model adjusted by: ^a^ Adjusted for age group, marital status, work regime, economic class, and race/color (R^2^ Nagelkerke values: Psychological = 0.05; Environmental = 0.098; Social = 0.021; Physical = 0.045; Global = 0.037). ^b^ Adjusted for age group, marital status, work regime, economic class, race/color, and diet (R^2^ Nagelkerke values: Psychological = 0.051; Environmental = 0.099; Social = 0.024; Physical = 0.046; Global = 0.037). ^c^ Adjusted for age group, marital status, work regime, economic class, race/color, diet, and nutritional status (R^2^ Nagelkerke values: Psychological = 0.064; Environmental = 0.104; Social = 0.024; Physical = 0.045; Global = 0.069). ^d^ Adjusted for age group, marital status, work regime, economic class, race/color, diet, nutritional status, and smoking (R^2^ Nagelkerke values: Psychological = 0.065; Environmental = 0.108; Social = 0.026; Physical = 0.045; Global = 0.069). ^e^ Adjusted for age group, marital status, work regime, economic class, race/color, diet, nutritional status, smoking, and drinking (R^2^ Nagelkerke values: Psychological = 0.065; Environmental = 0.108; Social = 0.031; Physical = 0.048; Global = 0.073). ^f^ Adjusted for age group, marital status, work regime, economic class, race/color, diet, nutritional status, smoking, drinking, and self-reported health (R^2^ Nagelkerke values: Psychological = 0.136; Environmental = 0.134; Social = 0.055; Physical = 0.112; Global = 0.280). ^g^ Adjusted for age group, marital status, work regime, economic class, race/color, diet, nutritional status, smoking, drinking, self-reported health, and physical activity (R^2^ Nagelkerke values: Psychological = 0.139; Environmental = 0.134; Social = 0.055; Physical = 0.113; Global = 0.297).

## Data Availability

The data presented in this study are available on request from the corresponding author on reasonable request.

## Abbreviations

QOL	Quality of life
WHOQOL	World Health Organization Quality of Life
WHO	World Health Organization
PA	Physical activity
SESI	Serviço Social da Indústria (Industrial Social Services)
BMI	Body mass index
